# Attachment sites of *Ixodes ricinus*, *Ixodes hexagonus*/*Ixodes canisuga* and *Dermacentor reticulatus* ticks and risk factors of infestation intensity and engorgement duration in dogs and cats

**DOI:** 10.1186/s12917-025-04535-z

**Published:** 2025-02-22

**Authors:** Julia Probst, Andrea Springer, Christina Strube

**Affiliations:** Institute for Parasitology, Centre for Infection Medicine, University of Veterinary Medicine Hannover, Buenteweg 17, 30559 Hanover, Germany

**Keywords:** Tick prophylaxis, Year-round tick protection, Tick detection, Tick attachment sites, Infestation risk

## Abstract

**Background:**

Over the last decades, climatic and environmental changes have led to an expanding seasonal activity pattern and increasing distribution of ticks across Europe. In particular, *Dermacentor reticulatus* is now commonly found on dogs in central Europe. The present study compared attachment sites between *Ixodes* spp. and *Dermacentor reticulatus* ticks collected by veterinarians from dogs and cats, and investigated risk factors associated with tick infestation intensity and engorgement duration.

**Results:**

The dataset comprised 6,335 dogs harbouring 10,287 ticks (8,095 *Ixodes ricinus*, 1,860 *D. reticulatus*, 218 *Ixodes hexagonus*/*Ixodes canisuga*, 114 of other tick species) respectively 4,248 cats harbouring 8,005 ticks (7,344 *I. ricinus*, 56 *D. reticulatus*, 505 *I. hexagonus*/*I. canisuga*, 100 of other tick species). Differing sites of tick attachment were not only found between the different host and tick species, but also between the tick developmental stages. Regarding the risk of infestation with multiple ticks, dogs and cats living in rural areas harboured significantly more often multiple than single specimens. Further, a long coat in cats was associated with a higher probability of multiple infestation, while this was not observed in dogs. However, there was a tendency towards a potential influence of the density of the undercoat (*p* = 0.051). In dogs, a tall to very tall body size as well as folded ears increased the risk of multiple infestation, while in cats, increasing age and increasing body size were negatively associated with multiple infestations. Ticks with an engorgement duration of > 48 h were found significantly more often on senior dogs and cats than on younger individuals, as well as on working/utility dog breeds, while engorgement duration was negatively correlated with infestation intensity in dogs. In cats, female gender and a rural residence were significantly associated with longer attachment duration.

**Conclusions:**

Individual as well as breed specific characteristics can lead to a higher tick infestation intensity or longer engorgement duration. The knowledge of tick attachment sites and specific risk factors can help to raise awareness among owners concerning the importance of tick control with licensed acaricides, as recommended e.g. by the European Scientific Counsel Companion Animal Parasites (ESCCAP), and may aid in early tick removal to decrease the risk of pathogen transmission to dogs and cats whose owners nonetheless refuse acaricidal drugs.

**Supplementary Information:**

The online version contains supplementary material available at 10.1186/s12917-025-04535-z.

## Background

Ticks transmit a variety of tick-borne pathogens (TBPs), causing tick-borne diseases (TBDs) in humans and animals (reviewed by 1). The most frequently detected tick species in Central Europe is the sheep or castor bean tick *Ixodes ricinus*, vector of *Anaplasma phagocytophilum*, *Borrelia* spp. and tick-borne encephalitis virus, among other TBPs [2]. The hedgehog tick *Ixodes hexagonus*, which is also a vector of *A. phagocytophilum* and *Borrelia* spp. [3, 4], is frequently found on dogs and cats as well [5], increasing the risk of TBP transmission. In addition, especially dogs are increasingly often infested with *Dermacentor reticulatus*, the ornate dog tick, which has dramatically expanded its range in Central Europe, e.g. in Poland [6], the Czech Republic [7] and Germany during the last decades [8, 9], and is the vector of *Babesia canis*, the principal cause of potentially life-threatening canine babesiosis in central Europe [10, 11].

Knowledge on the main attachment sites of different tick species is informative with regard to the TBP exposition risk, as ticks may be less or more conspicuous on different body parts. If the risk of tick infestation is minimal, e.g. in animals with restricted or no outdoor access, or if there are reservations about the use of acaricides, pet owners often opt for a regular adspectorial tick control [12], which can be improved by detailed knowledge on tick predilection sites. In dogs, the head and neck are often regarded as highly infested areas [13, 14], while for cats, less studies have been performed. In a study from the United Kingdom, the head (43% of the collected ticks), precisely the ears and the chin, as well as the neck (32% of the collected ticks) were the most commonly observed attachment sites of *I. ricinus* and *I. hexagonus* on cats, while on dogs both tick species were collected most frequently from the head (49% of the collected ticks). The remaining specimens were widely spread over the whole body surface with no further specific predilection sites [15]. A further study on cats in the USA focused mainly on tick species which are not native to Central Europe, like *Ambylomma americanum*, which was located especially in the anogenital area and the tail, as well as *Dermacentor variabilis* and *Ixodes scapularis*, which were mainly collected from head and back [16]. In a study from Australia on a comparably small number of 17 cats infested with *Ixodes holocyclus*, the head and ventral parts of the body like the axilla were described as highly infested [17].

The risk of TBP exposition may further be modulated by individual animal traits related to their breed or living conditions including its purpose. Knowledge on these risk factors may help to educate pet owners regarding the necessity of tick prophylaxis. Previous studies on host-specific characteristics associated with a higher tick infestation risk have predominantly focused on dogs infested with mainly *I. ricinus* as well as *I. hexagonus* or *D. reticulatus* [14, 18, 19]. Long haired and tall to very tall dogs were significantly more likely to be infested with *I. ricinus* and *D. reticulatus* in a study from Germany [14], as well as with *Rhipicephalus sanguineus* and *I. ricinus* in a study from Italy [18]. In another study from Great Britain, non-neutered dogs as well as working and utility breeds were significantly more often infested with first and foremost *I. ricinus* ticks, as well as with some *I. hexagonus*, *I. canisuga* and *D. reticulatus* specimens [19].

Besides the frequency of infestation, a long attachment duration is one of the driving factors of infection with different TBPs [20]. A previous study has determined average attachment durations of 78.8 h on dogs and 82.7 h on cats regarding female *I. ricinus*. Furthermore, 42.0% (487/1,159) of *D. reticulatus* specimens collected from dogs were classified as fully engorged in the same study [5]. Such very long periods of tick attachment allow the transmission of many different pathogens like *A. phagocytophilum*, *Borrelia* spp. or *B. canis*. Regarding *A. phagocytophilum*, transmission may already occur after six hours of feeding in an in vitro system under laboratory conditions, but an establishment of infection in dogs was only observed when ticks were attached for more than 48 h [21]. Another study in mice revealed that under natural circumstances, the transmission takes place around 36 h after the start of the feeding process [22]. Concerning *Borrelia* spp., it is commonly assumed that transmission of infectious spirochetes usually takes place after an attachment time of 24 h, although an earlier transmission to the host in less than 16 h is possible (reviewed by 23). For *B. canis*, sporogony in the tick salivary glands requires at least 48 h before transmission can take place [24, 25], although an earlier transmission after eight and 24 h has been observed for male *D. reticulatus* specimens in the case of an interrupted blood meal under laboratory conditions [26]. To the authors’ knowledge, no studies examining the influence of different host specific characteristics on the duration of tick engorgement in dogs and cats have been published to date.

In the present study, data from a large-scale tick submission study conducted in Germany and Austria during 2020 and 2021 were evaluated regarding tick attachment sites of the three most often collected tick species from dogs and cats. Further, factors that could in- or decrease the infestation intensity as well as the duration of engorgement were analysed to improve our knowledge on the tick infestation and TBP exposition risk of European dogs and cats.

## Methods

### Tick collection study

For this study, 219 veterinary practices from Germany and Austria collected ticks from privately owned dogs and cats between March 2020 and October 2021 as convenience sampling during routine clinical examinations [5]. A questionnaire was used to gather information about the host, including the species, sex, breed, length and structure of the hair coat as well as whether the animal was kept for a specific purpose or exhibited a specific outdoor behaviour like mousing. Additionally, the animal’s residence was characterized as urban, rural, coastal or limited to the own property. Further, each questionnaire contained two animal silhouettes– a dorsal and a ventral view– to mark the attachment site of every collected tick as precisely as possible (Additional Fig. 1).

**Fig. 1:**
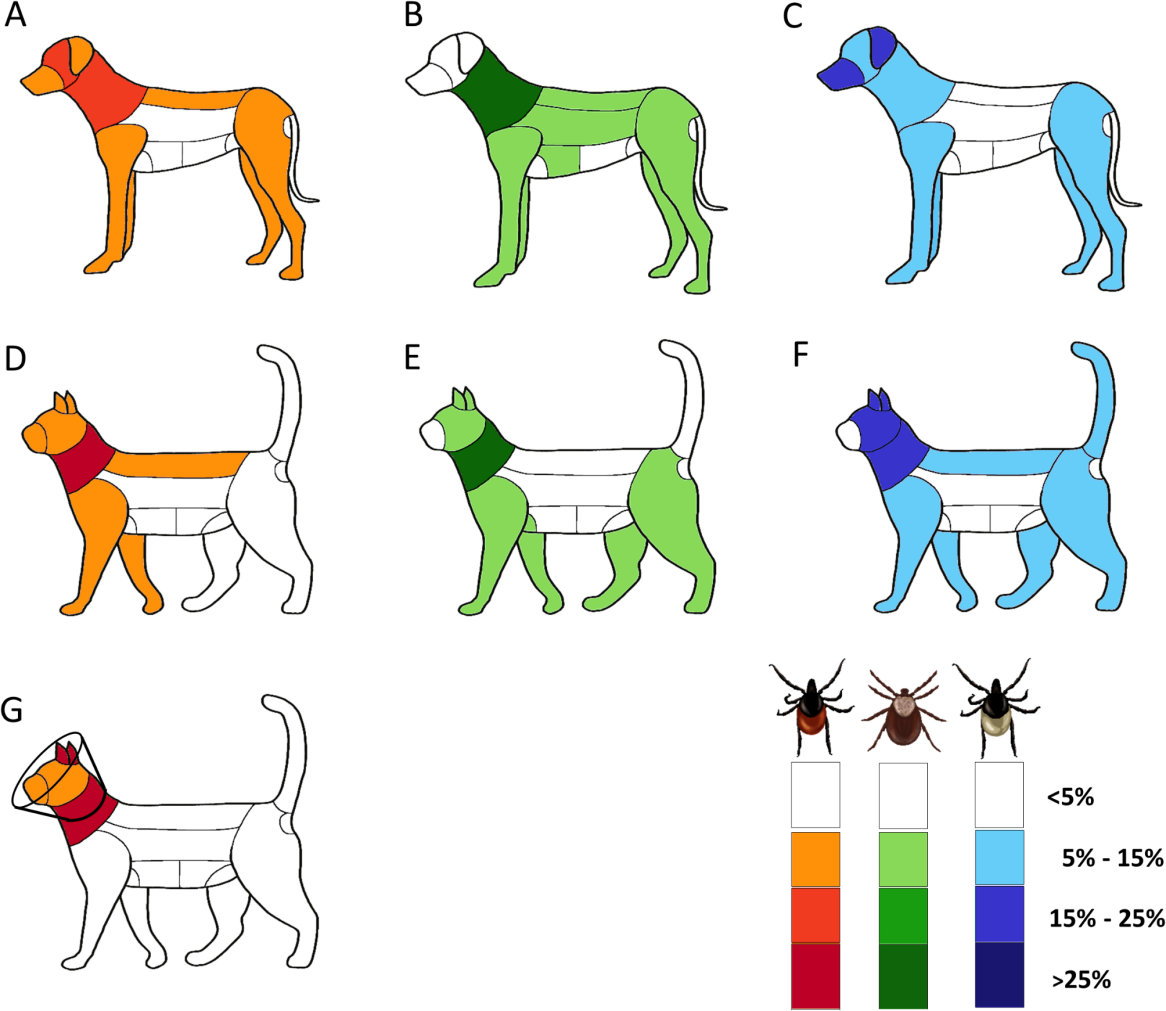
Distribution of tick attachment sites on dogs and cats sampled in the tick collection study for *I. ricinus* (**A**, **D**), *D. reticulatus* (**B**, **E**) and *I. hexagonus*/*I. canisuga* (**C**, **F**). The percentages refer to the total number of ticks for which the attachment site was documented, excluding free crawling ticks and those without a documented site of attachment. Further, the attachment sites of *I. ricinus* specimens from cats sampled in the frame of a clinical laboratory study are shown (**G**). Note that these cats were wearing a collar. The shading indicates the percentage of ticks recovered from the muzzle, ears, head, neck, dorsal and lateral rump, axillae, breast, abdomen, inguinal region, fore- and backlimbs, anogenital area and tail, respectively, with darker shades representing higher values

The submitted specimens were sent to the Institute for Parasitology, University of Veterinary Medicine Hannover, Germany, and were identified according to morphological keys [5]. Moreover, female *I. ricinus* specimens were measured using the OLYMPUS cellSens Entry (v. 3.2) software paired with an OLYMPUS SC50 camera adapter to determine the coxal index to estimate the attachment duration [5, 27].

### Data from experimentally tick infested cats

Within the frame of a previously performed clinical laboratory study (unpublished), which also included the infestation of cats with *I. ricinus* ticks, 24 European shorthair cats were experimentally infested two times at an interval of three months with 50 viable, adult and unfed specimens each. All cats were bred and owned by the Institute of Parasitology, University of Veterinary Medicine Hannover, Germany. Ticks from all cats were removed and counted 48 h after infestation. The sites of attachment were documented as additionally generated data, using the same drawings as in the questionnaire of the tick collection study (cf. Additional Fig. 1) to allow a direct comparison. During the 48 h of infestation, the cats wore a collar to prevent a removal of ticks from the head/upper neck region.

### Statistical analyses of tick attachment sites

The distribution of the tick attachment sites was compared between dogs and cats via Chi-square test in R. v. 4.1.1717 [28]. Furthermore, for each host species the attachment sites were compared between the different tick species as well as between adult and immature stages (nymphs and larvae) of *I. ricinus* and *I. hexagonus/I. canisuga* via Chi-square test or, in case of small sample sizes, Fisher’s exact test with simulated *P*-value (based on 10^6^ replicates).

### Data preparation: characterisation of breed-related features in the tick collection study

For purebred animals, breed specific characteristics were obtained from the respective standards of the Fédération Cynologique Internationale (FCI) for dogs and the Fédération Internationale Féline (FIFe) for cats. Concerning dogs, the body size was categorized as small (18–29 cm), medium (30–49 cm), tall (50–69 cm) and very tall (> 70 cm), while cats were only categorized as medium and tall according to their FIFe standard. While in cats the coat length was divided into short and long, in dogs also an average coat length was included. In addition, “partially coated” was introduced to include sampled Chinese Crested dogs. For both host species, the undercoat was classified into missing, moderately dense and dense. For dogs, the structure of the hair coat was classified into straight, coarse (rough and double-coated) and wavy/shaggy hair. Further, the shape of the dogs’ ears were defined as erect (prick, bat, candle flame and blunt-tip ears), folded (button, rose, V-shaped, cocked and drop ears) and dropping, describing ears which are hanging flat beside the head like a pendulum. The dog breed’s purpose was categorised according to the breed standards, their specific outdoor behaviour and the estimated time of contact with vegetation and natural surroundings. The six resulting categories were small and highly owner orientated toy breeds, companion breeds, greyhounds, hunting breeds and herding as well as working and utility breeds, which were combined into one category according to their similar outdoor behaviour.

### Statistical analyses of the tick collection study questionnaire data

Statistical analyses of the data obtained from the questionnaire of the tick collection study were performed using R. v. 4.1.1717 [28]. The influence of different predictors on the probability of being infested with multiple vs. a single tick was tested separately for dogs and cats in the subset of purebred animals by generalized linear mixed models (GLMM) with binomial error structure. Animal breed was used as a random factor, while fixed factors for dogs were age, as well as coat length and structure, density of the undercoat, shape of the ears, breed purpose and the character of the animal’s residence (urban vs. rural vs. only on the own property) as well as the tick collection month and year. For cats, age, gender, body size, coat length, the character of the animal’s residence (urban vs. rural vs. only on the own property) as well as the month and year of tick collection were included as fixed factors. For both cats and dogs, only few owners reported a coastal residence. Therefore, this category was excluded from statistical evaluation, but rather the information on a rural or urban residential character was used for these animals if stated additionally to “coastal”.

A second set of GLMMs was performed separately for dogs and cats to investigate risk factors associated with a long duration of engorgement. Since treating engorgement duration of female *I. ricinus* tick as a continuous response variable did not result in interpretable models, each tick was classified as having engorged ≤ 48 h vs. > 48 h to allow calculation of a binomial GLMM. The cut-off of 48 h was chosen based on the transmission dynamics of relevant TBPs, with a high likelihood of successful transmission after 48 h [21, 23, 26]. Included fixed factors were the age group, sex, body size, coat length, density of the undercoat, breed purpose, the animal’s infestation intensity and the character of the animal’s residence, while the animal’s identification number was treated as a random factor.

Non-significant predictors were sequentially removed from the model until no further improvement of the model fit as indicated by the corrected Akaike information criterion (AICc) was achieved [29]. The final models were compared with null models containing only the random factor using likelihood ratio tests (R-function “anova”). Final GLMMs were visualized by coefficient plots displaying estimates with standard errors.

## Results

In total, 18,292 ticks from dogs and cats were collected between March 2020 and October 2021, comprising 10,287 specimens collected from 6,335 dogs and 8,005 specimens from 4,248 cats. Due to the difficulty to differentiate between larval and nymphal *I. hexagonus* and *I. canisuga*, results for these two tick species were combined. From dogs, 8,095 specimens were identified as *I. ricinus*, 1,860 as *D. reticulatus* and 218 as *I. hexagonus*/*I. canisuga*, while 7,344 of the ticks collected from cats were identified as *I. ricinus*, followed by 56 *D. reticulatus* and 505 *I. hexagonus*/*I. canisuga* ticks. A further five tick species were identified, but were not included into evaluation of risk factors and tick attachment sites on species level due to their low number.

### Attachment sites in dogs

For 79.5% (8,178/10,287) of all collected ticks from dogs, the site of tick attachment was documented, whereas no attachment site information was available for 20.5% (2,109/10,287). The majority of ticks was collected from the head (2,883/8,178; 35.3%), thereof 28.9% (832/2,883) from the ears. Further ticks were collected from the rump (1,984/8,178; 24.3%), the neck (1,694/8,178; 20.7%) and the extremities (1,518/8,178; 18.6%). The detailed sites of attachment are shown in Additional Table 1.

**Table 1:** Number of ticks collected from dogs and cats in the tick collection study according to their area of attachment. Listed are the different developmental stages of the three most often collected tick species *I. ricinus*, *I. hexagonus* / *I. canisuga* and *D. reticulatus.* For *D. reticulatus*, only adult stages were collected

	I. ricinus			I. hexagonus / I. canisuga			D. reticulatus
	Adults	Nymphs	Larvae	Adults	Nymphs	Larvae	Adults
**Dogs**							
Ears	688/6,560 (10.5%)	2/65 (3.1%)	0/9 (0.0%)	27/123 (22.0%)	10/57 (17.5%)	0/7 (0.0%)	53/1,267 (4.2%)
Head (without ears)	1,914/6,560 (29.2%)	20/65 (30.8%)	2/9 (22.2%)	27/123 (22.0%)	17/57 (29.8%)	7/7 (100%)	78/1,267 (6.2%)
Neck	1,282/6,560 (19.5%)	11/65 (16.9%)	0/9 (0.0%)	17/123 (13.8%)	7/57 (12.3%)	0/7 (0.0%)	355/1,267 (28.0%)
Rump	1,513/6,560 (23.0%)	13/65 (20.0%)	0/9 (0.0%)	11/123 (8.9%)	10/57 (17.5%)	0/7 (0.0%)	422/1,267 (33.3%)
Frontlegs	602/6,560 (9.2%)	16/65 (24.7%)	6/9 (66.7%)	24/123 (19.5%)	6/57 (10.5%)	0/7 (0.0%)	204/1,267 (16.1%)
Hindlegs	501/6,560 (7.6%)	3/65 (4.6%)	1/9 (11.1%)	17/123 (13.8%)	7/57 (12.3%)	0/7 (0.0%)	117/1,267 (9.2%)
Other	60/6,560 (0.9%)	0/65 (0.0%)	0/9 (0.0%)	0/123 (0.0%)	0/57 (0.0%)	0/7 (0.0%)	38/1,267 (3.0%)
Total	6,560	65	9	123	57	7	1,267
**Cats**							
Ears	707/6,174 (11.5%)	23/92 (25.0%)	4/16 (25.0%)	32/202 (15.8%)	36/165 (21.8%)	10/21 (47.6%)	3/48 (6.3%)
Head (without ears)	1,265/6,174 (20.5%)	29/92 (31.5%)	1/16 (6.3%)	21/202 (10.4%)	56/165 (33.9%)	1/21 (4.8%)	8/48 (16.7%)
Neck	2,848/6,174 (46.1%)	20/92 (21.7%)	1/16 (6.3%)	54/202 (26.7%)	16/165 (9.7%)	1/21 (4.8%)	18/48 (37.5%)
Rump	696/6,174 11.3%)	9/92 (9.8%)	4/16 (25.0%)	35/202 (17.3%)	24/165 (14.5%)	1/21 (4.8%)	7/48 (14.6%)
Frontlegs	494/6,174 (8.0%)	7/92 (7.6%)	4/16 (25.0%)	19/202 (9.4%)	11/165 (6.7%)	7/21 (33.3%)	6/48 (12.5%)
Hindlegs	103/6,174 (1.7%)	3/92 (3.3%)	0/16 (0.0%)	22/202 (10.9%)	16/165 (9.7%)	0/21 (0.0%)	4/48 (8.3%)
Other	61/6,174 (1.0%)	1/92 (1.1%)	2/16 (12.5%)	19/202 (9.4%)	6/165 (3.6%)	1/21 (4.8%)	2/48 (4.2%)
Total	6,174	92	16	202	165	21	48

Concerning *I. ricinus*, the attachment site was not documented for 16.4% (1,324/8,095) of the specimens, while 1.7% (137/8,095) were not yet attached but found crawling on the host. Nevertheless, for the majority of specimens the attachment sites were documented (6,634/8,095; 82.0%). Most of these were detached from the head (2,626/6,634; 39.6%), followed by 23.0% (1,526/6,634) from the rump, 19.5% (1,293/6,634) from the neck and 17.0% (1,129/6,634) from the extremities, as well as 0.9% (60/6,634) from the tail, anogenital region and other body areas (Fig. 1). Further, there were significant differences in the distribution of tick attachment sites between adult and immature stages (Fisher’s Exact test, *P* < 0.001). While adults were first and foremost collected from the head (2,602/6,560; 39.7%), immature stages were attached to the head and frontlegs in almost equal proportions (24/74 and 22/74; 32.4% and 29.7%). Although differences between nymphs and larvae are difficult to assess due to the low number of collected larvae, a detailed overview of the stage-specific attachment sites is given in Table 1.

Concerning *I. hexagonus*/*I. canisuga*, the site of attachment was not documented for 13.8% (30/218) of the collected ticks, while 0.5% (1/218) were found crawling on the host. For 85.8% (187/218), information on the attachment site was available, with most of the ticks collected from the ears (37/187; 19.8%) and the muzzle (21/187; 11.2%). Further specimens were found on the extremities (54/187; 28.9%), the neck (24/187; 12.8%), the rump (21/187; 11.2%), as well as 16.0% (30/187) on further parts of the head (Fig. 1). While adult ticks were located on the ears and other parts of the head in equal proportions (27/123 each; 22.0%), immature stages were mainly collected from other parts of the head (24/74; 32.4%) (Table 1), however, this difference was not statistically significant (Fisher’s Exact test, *P* = 0.081).

For 29.8% (555/1,860) of the *D. reticulatus* specimens, no site of attachment was documented and 2.0% (38/1,860) were found crawling on the host, while attachment site information was available for 68.1% (1,267/1,860) of the ticks. The overall distribution differed significantly compared to *I. ricinus* (χ^2^ = 436.62, df = 4, *P* < 0.001) and *I. hexagonus/I. canisuga* (Fisher’s Exact test, *P* < 0.001). Most of the attached *D. reticulatus* were collected from the rump (422/1,267; 33.3%), followed by 28.0% (355/1,267) from the neck and 25.3% (321/1,267) from the extremities. The head was infested by 10.3% (131/1,267) of the collected *D. reticulatus* specimens, of which 40.5% (53/131) were located on the ears, while further ticks were attached to the tail, the anogenital region and other body areas (38/1,267; 3.0%) (Fig. 1). Because only adult specimens were collected, no differences between developmental stages can be reported.

### Attachment sites in cats

For 85.0% (6,807/8,005) of all ticks collected from cats, the site of attachment was documented, whereas for 15.0% (1,198/8,005) of the ticks, no site of attachment was given. Further, the distribution differed significantly to the observations made on ticks from dogs (χ^2^ = 1239.3, df = 4, *P* < 0.001). Combining all tick species, the largest proportion was collected from the neck (3,049/6,807; 44.8%), followed by the head (2,238/6,807; 32.9%), with 39.7% of those (889/2,238) detached from the ears. Further ticks were detached from the rump (725/6,807; 10.7%) and the extremities (701/6,807; 10.3%).

Similar to dogs, most of the collected ticks were identified as *I. ricinus*. For 14.0% (1,028/7,344) of all *I. ricinus* collected from cats, no site of attachment was recorded and 0.5% (34/7,344) were crawling on the host. Of the remaining 85.5% (6,282/7,344) with documented attachment site, 45.7% (2,869/6,282) were detached from the neck, followed by the head (2,029/6,282; 32.3%), the rump (709/6,282; 11.3%) and the extremities (611/6,282; 9.7%), while 64 ticks were located on other body areas (1.0%). Of the ticks collected from the head, 36.2% (734/2,029) were located in the ear region (Fig. 1). This distribution differed significantly to the distribution of *I. ricinus* ticks on dogs (χ^2^ = 1074.9, df = 4, *P* < 0.001). Moreover, significant differences were also observed with regard to the attachment sites of adult and immature *I. ricinus* (Fisher’s Exact test, *P* < 0.001), as adults were mainly collected from the neck (2,848/6,174; 46.1%), while immature stages (nymphs and larvae) were primarily located at the ears (27/108; 25.0%) and other parts of the head (30/108; 27.8%), as shown in detail in Table 1.

Concerning the attachment sites of adult *I. ricinus* specimens documented as additional data in the frame of the previously performed clinical laboratory study, these differed significantly to those of *I. ricinus* specimens collected from naturally infested cats (χ^2^ = 359.07, df = 4, *P* < 0.001). Most of the ticks were collected from the head (525/848; 61.9%), whereof 60.2% (316/525) were attached on, in or around the ears, followed by the neck (242/848; 28.5%), the tail (29/848; 3.4%), the rump (26/848; 3.1%) and the extremities (22/848; 2.6%) (Fig. 1). More detailed sites of attachment observed in the laboratory study are listed in Additional Table 2.

**Table 2:** Number of tick infested dogs and cats according to their individual or breed related hair coat characteristics. Note that the structure of the hair coat was not analysed in cats

	Dogs	Cats
**Hair length**		
Short	1,347/6,335 (21.3%)	3,375/4,248 (79.5%)
Average	1,527/6,335 (24.1%)	27/4,248 (0.01%)
Long	1,074/6,335 (17.0%)	323/4,248 (7.6%)
Partially coated	6/6,335 (0.01%)	0/4,248 (0.0%)
Unknown	2,386/6,335 (37.7%)	525/4,248 (12.4%)
**Structure of the hair coat**		
Straight	3,097/6,335 (48.9%)	NA
Coarse	661/6,335 (10.4%)	NA
Wavy/Shaggy	368/6,335 (5.8%)	NA
Unknown	2,214/6,335 (35.0%)	NA
**Density of the undercoat**		
Missing	1,015/6,335 (16.0%)	4/4,248 (0.01%)
Moderate	516/6,335 (8.2%)	3,452/4,248 (81.3%)
Dense	2,595/6,335 (41.0%)	269/4,248 (6.3%)
Unknown	2,214/6,335 (35.0%)	525/4,248 (12.4%)

NA = not applicable

Regarding *I. hexagonus/I. canisuga* collected from cats, the documented tick attachment sites differed significantly to those of *I. ricinus* (χ^2^ = 194.85, df = 4, *P* < 0.001). For 23.2% (117/505) of the ticks, no attachment site was documented, while no free crawling ticks were recorded. The 388 specimens for which a site of attachment was documented (76.8%) were primarily collected from the head (156/388; 40.2%), followed by the neck (71/388; 18.3%), the rump (60/388; 15.5%) and the extremities (75/388; 19.3%), while 26 ticks were detached from other body parts (6.7%). Of those collected from the head, 50.0% were located in the ear region (78/156) (Fig. 1). The distribution differed significantly between adult and immature stages (χ^2^ test, χ^2^ = 46.32, df = 6, *P* < 0.001), as adults were mostly detached from the neck (54/202; 26.7%), while immature stages were mainly located on the head (57/186; 30.6%), followed by the ears (46/186; 24.7%), shown in detail in Table 1.

Concerning *D. reticulatus*, only few specimens (56/8.005; 0.7%) were collected from cats, whereof 85.7% (48/56) had a documented attachment site, while no attachment site was documented for 14.3% (8/56). The ticks were primarily collected from the neck (18/48; 37.5%), followed by the extremities (10/48; 20.8%), the head (11/48; 22.9%) with three of these specimens collected from the ears (27.3%), the rump (7/48; 14.6%) and other parts of the body (2/48; 4.2%) (Fig. 1 and Additional Table 1). The recorded tick attachment sites of *D. reticulatus* specimens differed significantly to those of *I. ricinus* (Fisher’s Exact test, *P* = 0.02) and *I. hexagonus*/*I. canisuga* (Fisher’s Exact test, *P* = 0.03). As in dogs, only adult specimens were collected and no differences between developmental stages can be reported.

### Risk factors in dogs

**Fig. 2:**
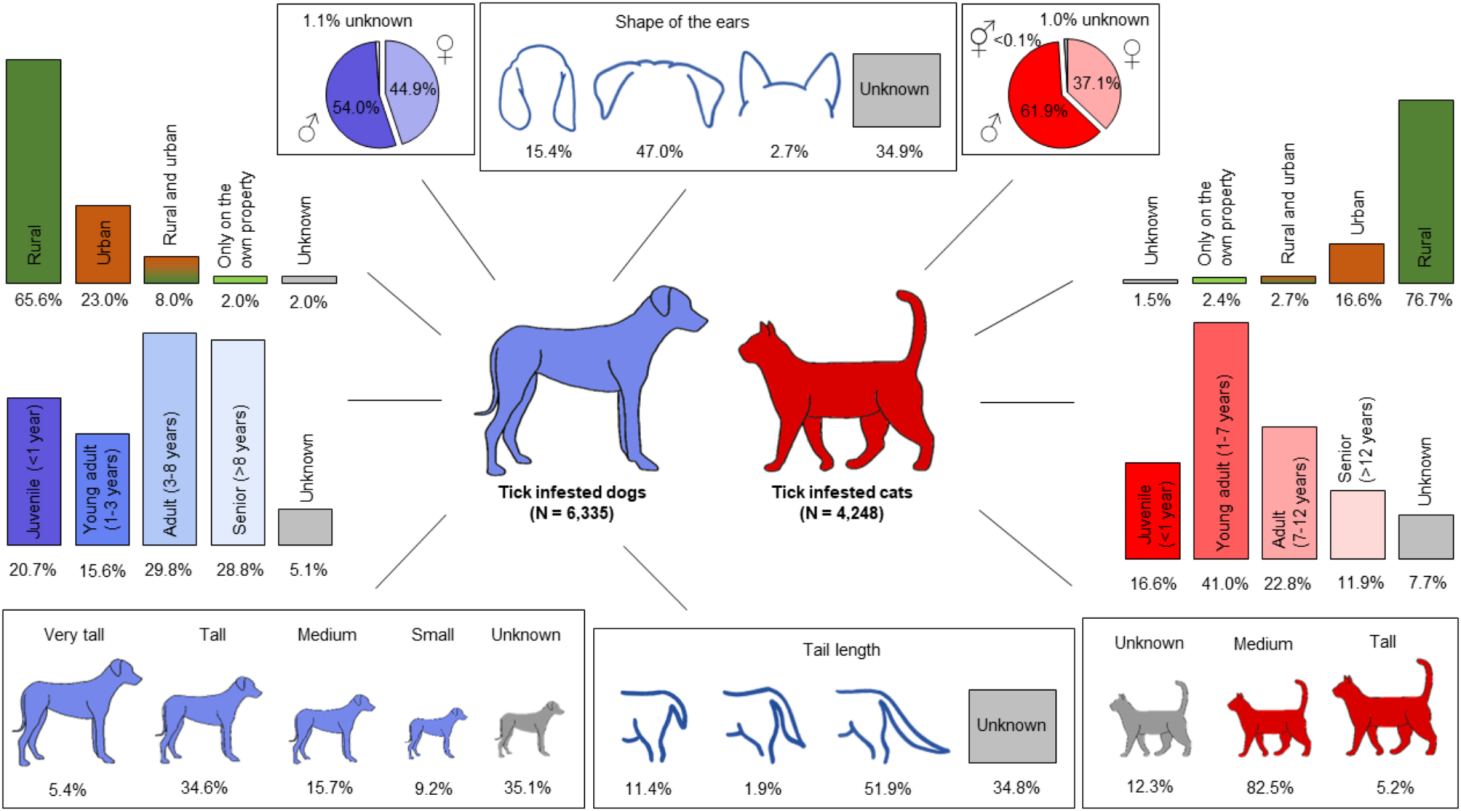
Percentage distribution of intrinsic (gender, age, body height, shape of the ears and tail length) and extrinsic (residence) characteristics of tick infested dogs and cats included in the study. For cats, different ear shapes or tail lengths were not considered due to little variability

The sex ratio of the sampled dogs was approximately even with 54.0% males and 44.9% females, including 39.6% (2,507/6,335) neutered animals. Of the 3,423 males, 36.4% were neutered (1,247/3,423), 63.4% were intact (2,169/3,423) and for 0.2% of the animals no information was given (7/3,423). Of the female dogs, 43.9% (1,247/2,841) were neutered, 56.1% (1,593/2,841) were intact and for one dog the castration status was unknown. The average age was 6.6 years and most of the sampled animals were adult dogs between one and eight years (2,876/6,335; 45.4%) (Fig. 2). Of the 6,335 dogs, 1,587 (25.1%) belonged to hunting breeds from which 227 (14.3%) were specifically described as dogs used for hunting. Companion breeds were represented by 1,406 (22.2%) dogs, of which 541 (38.5%) belonged to small and highly owner-orientated toy breeds. Further, 579 (9.1%) dogs were of herding breeds, with only 8 (1.4%) dogs actively herding, 508 (8.0%) of working and utility breeds, with 75 (14.8%) of them actively used, and 46 (0.7%) of greyhound breeds. For 2,214 (35.0%) dogs, no distinct breed was mentioned. Further, 1.1% (69/6,335) of the dogs were described as mice or rat hunters. Hair coat characteristics like hair length, structure of the hair coat as well as the density of the undercoat in infested dogs are described in detail in Table 2.

**Fig. 3:**
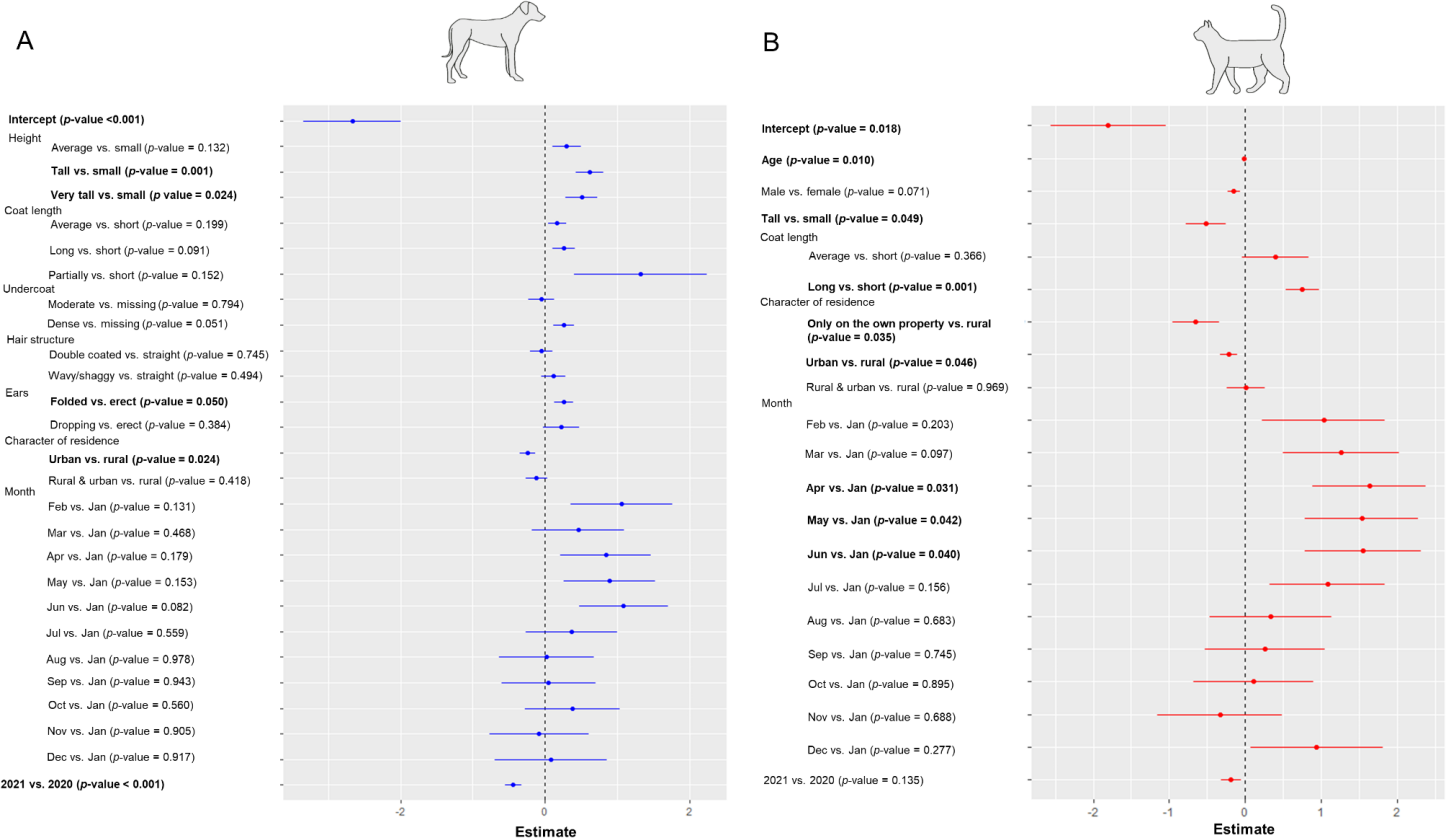
Coefficient estimates with standard errors of the binomial GLMM investigating the effect of different predictor variables on the tick infestation type (single vs. multiple ticks) in dogs (**A**) and cats (**B**) for which breed information was available (*N* = 3,648 dogs, *N* = 3,173 cats). Significant *P*-values are printed in bold. The models were significantly different from null models containing only the dog respectively cat breed as a random factor (A: Chi-square = 109.21, Df = 26, *P* < 0.001; B: Chi-square = 152.37, Df = 20, *P* < 0.001)

The final GLMM for tick infestation intensity, including 3,684 dogs with breed information, contained the factors body size, coat length and structure, density of the undercoat, shape of the ears, the character of the animal’s residence (urban vs. rural) as well as the tick collection month and year (Fig. 3 and Additional Table 3), while the non-significant factors age, sex, castration status, tail length and breed purpose were sequentially excluded to improve the model fit. The model showed that dogs from tall and very tall breeds were significantly more often infested with multiple ticks than small breeds, while no significant difference between small and medium sized breeds was observed. Further, no significant influence of the coat length and density of the undercoat was apparent, although a *p*-value of 0.051 indicated a tendency towards a possible positive influence of a dense undercoat. Additionally, an urban residence was associated with a lower probability of multiple infestation compared to a rural residence. No monthly differences were observed, but significantly less dogs were multiple-infested in the year 2021 compared to 2020 (Fig. 3 and Additional Table 3).

**Fig. 4:**
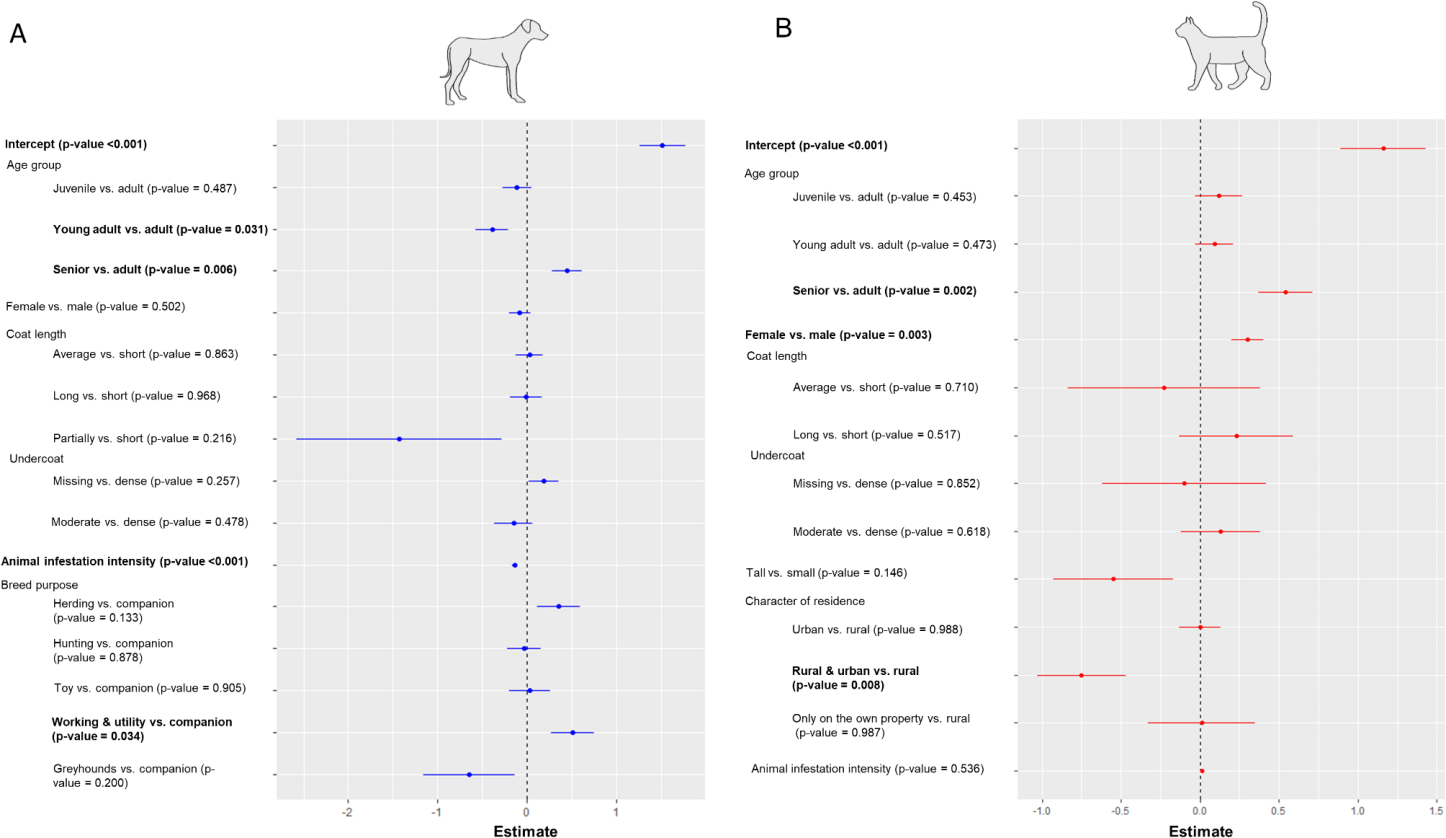
Coefficient estimates with standard errors of the binomial GLMM investigating the effect of different predictor variables on the duration of engorgement (≤ 48 h vs. > 48 h) of ticks collected from dogs (**A**) for which breed information was available (*N* = 2,101), and from cats (**B**), including pure and mixed breed animals (*N* = 2,583). Significant *P*-values are printed in bold. The models were significantly different from null models containing the dog breed and animal ID respectively cat’s ID as a random factor (A: Chi-square = 78.17, Df = 15, *P* < 0.001; B: Chi-square = 33.69, Df = 13, *P* = 0.001)

The final GLMM for the engorgement duration, based on 2,593 ticks from 2,101 animals with breed information, included the factors age group, sex, coat length, density of the undercoat, breed purpose and the animal’s infestation intensity (Fig. 4 and Additional Table 4). Host body size, castration status, coat structure, ear and tail characteristics, character of residence as well as month and year were non-significant and their exclusion improved model fit. The model showed a higher probability of an engorgement duration > 48 h for ticks from senior dogs, respectively a shorter engorgement duration in ticks from young adult compared to adult dogs. Concerning the different breeds, the GLMM showed a longer engorgement duration in ticks from working and utility dogs compared to companion breeds. Further, no significant influence of animal sex, coat length and undercoat density was observed, while the animal’s infestation intensity was negatively associated with engorgement duration (Fig. 4 and Additional Table 4).

### Risk factors in cats

Of the sampled cats, 61.9% (2,630/4,248) were males and 37.1% (1,574/4,248) females, while one cat was described as a hermaphrodite. Overall, 75.2% (3,194/4,248) were neutered, with 77.0% (2,025/2,630) neutered and 23.0% (605/2,630) intact male cats, and 73.8% (1,161/1,574) neutered and 26.0% (409/1,574) intact female cats. For four female cats, no castration status was given. The average age of the infested cats was 6.7 years, with young adults from one to 7 years representing 41.0% of sampled cats (1,743/4,248) (Fig. 2). Regarding the length of the hair coat, more short (3,375/4,248; 79.5%) than long haired cats (323/4,248; 7.6%) were part of the study (Table 2), in line with the fact that 75.7% (3,216/4,248) of the animals were European short hair cats, representing the most common breed in Germany. Other breeds were represented by 12.0% (509/4,248) of the cats, while for further 12.4% (525/4,248) no breed was specified. Further, information on the density of the undercoat in infested cats are given in Table 2.

The final GLMM for tick infestation intensity included 3,173 animals and contained the factors age, sex, body height, coat length, the character of the animal’s residence (urban vs. rural) as well as the tick collection month and year, while castration status was excluded. The density of the undercoat, the structure of the hair coat and the shape of the ears showed low variability among the sampled cats and were for this reason not included in the final GLMM, but are listed in Table 2. The model showed a significantly higher probability of carrying multiple ticks for animals with a long hair coat compared to short coated ones, while tall cats were significantly less often multiple infested than small individuals (Fig. 3 and Additional Table 3). Further, a significant negative effect of cat age on infestation intensity was apparent, as multiple infestations were less often detected with increasing age. Cats living in an urban area or only on the owner’s property had a significantly lower probability of multiple infestation than rural cats. Additionally, multiple infestations occurred significantly more often during April to June compared to January, while, in contrast to dogs, no annual difference was apparent.

The final GLMM for engorgement duration of 3,859 female *I. ricinus* ticks from 2,583 cats contained the factors age group, sex, body size, coat length, density of the undercoat, the animal’s infestation intensity and the character of the animal’s residence. Similar to the model for dogs, castration status as well as collection month and year were excluded to improve model fit. The model showed a higher probability of an engorgement duration > 48 h for ticks from female compared to male cats and from senior compared to adult cats. A mixed residential character (urban and rural parts) was negatively associated with engorgement duration. Similar to the GLMM for ticks from dogs, no significant influence of coat length nor density of the undercoat or body size was apparent. In contrast to ticks from dogs, the cats’ infestation intensity was also not significantly associated with the duration of engorgement (Fig. 4 and Additional Table 4).

## Discussion

The aim of the present study was to describe attachment site patterns of the most relevant tick species of dogs and cats in central Europe and to assess risk factors for tick infestation intensity and tick engorgement duration. The European tick fauna has recently undergone a dramatic change due to the spread of *D. reticulatus* [8, 9, 30], which now constitutes more than 50.0% of ticks collected from dogs in some regions of Germany [5]. Most previous studies on risk factors related to tick infestation and/or tick attachment sites have been published more than ten years ago [13] or have investigated tick species which have no relevance in Central Europe [16, 17, 31]. Moreover, the only study performed in Germany was limited to dogs [14]. Therefore, this is the first study evaluating risk factors of tick infestation for cats in Germany. One limitation of the study was that only tick-infested animals were sampled, while information on tick-free animals was not available, precluding risk factor analysis of tick infestation per se. Therefore, risk factors were analysed with regard to the probability of carrying multiple ticks vs. a single tick and with regard to engorgement duration. To the authors’ knowledge, no previous studies on risk factors related to tick engorgement duration have been published so far, despite the importance of this aspect for TBP transmission. No information on acaricidal treatment of the animals was obtained, however, as all of them carried ticks, it is evident that they were not or not completely protected against infestation. Although it cannot be excluded that partial protection may have affected some of the results, this was expected and the varying status of acaricidal protection was regarded as representative for the general pet population.

### Attachment sites in dogs and cats

Detailed information on tick attachment sites may help to reduce the time of engorgement and thus the risk of TBD transmission when owners refuse to use licensed acaricidal drugs for tick control. Another major benefit is that veterinarians can specifically target these regions as part of veterinary examinations to inform owners about the risk of tick infestation and sensitise them for an effective acaricidal tick prophylaxis. Regarding the results of the present tick submission study, it should be kept in mind that the ticks were collected during veterinary examinations, thus, ticks previously detected and detached by the owners as well as ticks removed by the animals themselves were not included. This probably skewed the distribution towards regions of the animal’s body that are less often touched, less accessible or where ticks are less visible, but at the same time highlighting those areas where ticks are often overseen. Moreover, in cats, a comparison between attachment sites recorded in the tick submission study with those of cats experimentally infested with *I. ricinus*, wearing a collar to prevent tick removal, was possible.

Distinct differences in the attachment site distribution were observed between the two host species, most likely due to their different tick species composition. Previous studies have identified varying predilection areas for different tick species infesting dogs, for example the head for *I. ricinus* [13–15], the rump and the neck for *D. reticulatus* [14, 32] and the ear region respectively the interdigital spaces for *R. sanguineus* [18, 31]. In the present tick submission study, a third of the *I. ricinus* specimens from dogs were located on the head, followed by the rump and neck. Due to the large share of this tick species among the total number of ticks, this was the predominant tick distribution pattern on dogs in general. On the other hand, *D. reticulatus* specimens were mainly collected from rump, neck and extremities, in line with results from another German study, reporting that *D. reticulatus* specimens were significantly less often detached from the head compared to the back [14]. A study from Austria suggested that highly infested areas may be coherent with the arrival points of questing ticks on their host [13]. Questing of *I. ricinus* has been observed at an average height of 55 cm, while *D. reticulatus* showed an average questing height of 66 cm [33]. As dogs often carry their head lower than their back during sniffing, this might explain the different predilection areas of the two tick species, head and neck for *I. ricinus* and rump as well as back for *D. reticulatus*. Further, differing attachment sites of adult and immature stages of *I. ricinus* on both dogs and cats and of *I. hexagonus*/*I. canisuga* on cats were noted. Similar differences in attachment sites have already been reported for the different developmental stages of *I. ricinus* as well as *R. sanguineus* [34–36]. On red deer, *I. ricinus* larvae were mainly found on the legs and ears, nymphs on the ears and adults on the neck and in the groin. The authors suggested a short moving distance of immature stages as a main reason for these differences [36]. However, the smaller size of nymphs and larvae likely also influenced this distribution pattern, as they are less conspicuous to the owner than adult specimens, e.g. on the head, where adult ticks may be easily spotted. Nevertheless, it should be kept in mind that the sample size of nymphs and especially larvae of *I. ricinus* and *I. hexagonus*/*I. canisuga* was comparably low in the present study, so that further investigations are necessary to evaluate the recent results.

In cats, almost half of the ticks collected in the submission study were detached from the neck followed by the head, confirming the neck as a typical attachment site for the two most frequently collected tick species from cats in this study, *I. ricinus* and *I. hexagonus/I. canisuga*, for which a similar distribution pattern was already reported in a study from Great Britain [15]. Regarding *I. ricinus* in cats, the results of the tick submission study were compared to a clinical laboratory study, which more closely reflected the natural predilection sites by excluding a bias due to tick removal by the owner. Moreover, the cats wore a collar to prevent tick removal by self-grooming. In the laboratory study, a higher proportion of ticks was collected from the head compared to the field study. The ears were highly infested, especially related to their very small proportion of the total body surface, with over a third of the collected ticks. This confirms the ears as a preferred attachment site of *I. ricinus* compared to other parts of the head [15], as previously noted in dogs [13, 14, 18]. Under natural conditions, ticks located on the ears are probably easily detected and removed by the owner or the cat itself by self-grooming or scratching, which was prevented by the collar in the laboratory approach, explaining the difference between the laboratory and field study.

The *I. hexagonus/I. canisuga* specimens from the tick submission study were found first and foremost on the head of cats, also predominantly located at the sparsely haired and well perfused ear region, while the rest of the collected specimens were distributed all over the body in almost equal percentages. The head of cats is the first site of contact with nests of hedgehogs, the main hosts of *I. hexagonus*, which may be the reason for this predilection area [37].

Apart from differences between tick species, different patterns of human-animal contact and grooming of cats and dogs may have influenced the results. While cats are touched especially often on the head, dogs are more accepting of close and extended body contact and are therefore often groomed over the whole body. These differences may influence the frequency of tick removal from different body areas and might have affected the described patterns.

### Risk factors in dogs and cats

Together with the information about the tick attachment sites, knowledge on individual and breed specific factors that might increase the risk of multiple infestations or the duration of tick engorgement may help to improve regular tick prophylaxis by the owners. The modelling results showed distinct differences between dogs and cats with regard to host specific risk factors for the probability of multiple infestation. A previous study from Germany already reported a significant positive correlation of dog body size and tick infestation, especially in very tall breeds which were 9.7 times more likely infested with ticks than small dogs [14]. A similar correlation between the probability of multiple infestation and dog body size was observed in the present study. Further, a long hair coat was positively correlated with the number of ticks in previous studies [14, 18, 19]. In the present study, this was not observed for dogs, but for cats. A long hair coat generally makes it harder for the owner to detect and remove ticks [18]. Nevertheless, long-haired dogs might be groomed more frequently by the owner than free ranging long-haired cats, so that multiple infestations as well as a long tick attachment time might be prevented by early detections. However, no significant influence of the cats’ coat length on tick engorgement duration was detected, implying that the long hair predominantly impairs detection of ticks during the early phase of attachment. European short hair cats are often free ranging and less owner oriented compared to pure breeds with a longer hair coat like Maine Coons or Persians, which additionally require regular grooming. This behavioural difference might compensate for the effect of coat length, as short-haired cats might be less often handled and thereby examined for ticks by their owners. This could also explain the fact that a tall body size of cats was negatively correlated with the intensity of infestation, as these pure breeds are often large cats. Nevertheless, maybe less the hair length than the structure of hair and undercoat is relevant concerning the intensity or duration of tick infestation. A dense undercoat may impair the detection of ticks in dogs, resulting in more multiple infestations, although only a tendency was observed in the GLMM (*p* = 0.051), while a double coated hair might additionally complicate tick detection. Further, folded ears were positively correlated with the intensity of infestation compared to erect ears of dogs. This might be related to a better visibility of ticks in dogs with erect ears. Dropping ears were significantly negatively associated with intensity of infestation, possibly due to a higher awareness and intensity of care of the owner to prevent ear infections by regular cleaning.

In addition to body size and hair length, an American study also reported that young and male dogs had a higher risk of tick infestation [38], while no differences between the sexes were noted in another study from Germany [14]. The present study indicated a higher infestation intensity in younger than in older cats, which was not apparent for dogs. However, ticks collected from old animals of both host species had a significantly higher probability of having engorged for > 48 h, translating to a high likelihood of TBP transmission in case of infected ticks. Young individuals are known to be more explorative and active, which may increase their infestation risk, whereas less self-grooming or a less intensive contact with the owner may increase the engorgement duration in older animals.

Moreover, both dogs and cats from urban areas had a lower probability of multiple infestation, similar to results of other studies [18, 19, 39]. In urban areas, animals, particularly cats, are less likely to have unrestricted outdoor access, reducing their contact rate with tick habitats that are also less abundant in urban areas. A study from the UK described the frequency of exposure rather than the route or duration of the outdoor activity as the biggest risk factor of tick infestation [40]. In line with this, we would have expected herding and hunting dogs to carry significantly more ticks than companion or toy breeds, as already described in some previous studies [19, 38]. However, breed purpose showed no significant effect on infestation intensity in dogs and was removed from the final model. Nevertheless, it should be kept in mind that only a small proportion of the dogs was listed as actively herding or hunting, so that the actual risk of infestation in these categories might be lower than expected. However, a significantly longer engorgement duration was observed in working and utility breeds compared to companion breeds like previously reported [19]. A longer attachment duration could be driven by the fact that working/utility breeds often spend more time outdoors or in kennels, with less intensive contact to the owner than companion breeds.

Seasonal differences in the frequency of multiple infestations in this dataset have already been described by Probst et al. 2023 [5], wherefore sampling month was included in the statistical models of the present study. The observed differences between dogs and cats are probably driven by their different tick species composition. In both sampled host species, *I. ricinus* was the most often collected tick species, accounting for over 78.69% of all collected ticks from dogs and even 91.74% of all specimens collected from cats. While *D. reticulatus* was the second most common tick species in dogs, *I. hexagonus*/*I. canisuga* was the second most frequent tick species in cats [5]. The fact that no significant monthly differences were noted with regard to infestation intensity in dogs is probably caused by the complementary activity patterns of *I. ricinus* and *D. reticulatus*, resulting in a similar risk of multiple infestation year-round [5], i.e. also a risk of infestation over the winter months. Cats had a higher probability of harbouring multiple ticks from April to June, which covers the main activity period of *I. ricinus* and *I. hexagonus/I. canisuga* [41–43], and is in line with the distinct peak in multiple infestations with *I. hexagonus* in May and June [5].

## Conclusions

This large-scale study showed that most ticks were concentrated on the head and neck of both dogs and cats, however, the share of ticks on other body parts should also not be neglected. While knowledge on the main attachment sites is helpful to facilitate early tick removal, tick and consequently TBD prophylaxis relying solely on visual control for ticks can only be recommended if the risk for tick infestation is minimal. This risk is modulated by several intrinsic and extrinsic traits, such as characteristics of the hair coat, body size, age, sex and the animal’s surroundings, affecting the probability of multiple infestation and the duration of engorgement by an increased tick exposure or via impairment of tick detection and removal. Nevertheless, in dogs the coat length might be less relevant than previously described, as no influence on infestation intensity or engorgement duration was observed, proving an infestation risk independent from the length of the hair coat. Therefore, the use of licensed acaricides is recommended for all animals with outdoor access to achieve a comprehensive protection, not only to protect individual animals, but also to protect owners from ticks detached in the home environment and to prevent a further spread of potentially life threatening TBPs such as *B. canis*.

## Electronic supplementary material

Below is the link to the electronic supplementary material.

Supplementary Material 1

Supplementary Material 2

Supplementary Material 3

Supplementary Material 4

Supplementary Material 5

## Data Availability

Data is provided within the manuscript or supplementary information files.

## Abbreviations

AIC: Akaike information criterion
FCI: Fédération Cynologique Internationale
FiFe: Fédération Internationale Féline
GLMM: Generalized linear mixed models
TBD: Tick borne disease
TBP: Tick borne pathogen
