# Supplementary material for: Attachment sites of Ixodes ricinus, Ixodes hexagonus/Ixodes canisuga and Dermacentor reticulatus ticks and risk factors of infestation intensity and engorgement duration in dogs and cats
Source: BMC Vet Res. 2025 Feb 22;21:83. doi: 10.1186/s12917-025-04535-z (PMC11846248; doi:10.1186/s12917-025-04535-z)
Supplement: Supplementary file 1 — Supplementary Material 1 [file 12917_2025_4535_MOESM1_ESM.pdf]

# THE TICK QUESTIONNAIRE

| | |
|---|---|
| ID OF THE QUESTIONNAIRE | XXXXXX |
| ID OF THE TICK COLLECTION CONTAINER | XXXXXX |

! Please fill in all fields in block capitals!

1. MONTH OF TICK COLLECTION: ☐ 2020 ☐ 2021

2. COUNTRY AND POSTAL-CODE OF THE ANIMAL: COUNTRY POSTAL CODE

3. ANIMAL SPECIES: ☐ DOG ☐ CAT ☐ OTHER: ____

4. HOST ANIMAL SEX: ☐ MALE / ☐ NEUTRED
☐ FEMALE / ☐ NEUTRED

5. BREED:

6. AGE: ☐ YEARS ☐ MONTHS

7. CHARACTER OF THE HAIR COAT: ☐ LONG ☐ SHORT ☐ CURRENTLY CLIPPED
☐ OTHER: ____

8. WHERE WAS THE TICK LOCATED ON THE ANIMAL?

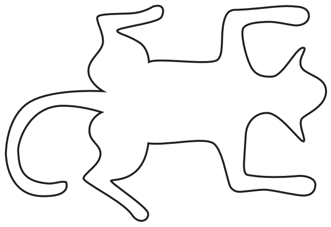

DORSAL

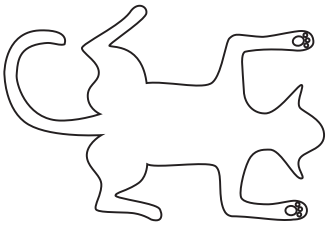

VENTRAL

9. WHERE HAS THE ANIMAL BEEN DURING THE LAST TWO WEEKS? ☐ URBAN AREA ☐ RURAL AREA
☐ COASTAL AREA ☐ OTHER

10. DOES THE ANIMAL HAVE A SPECIAL PURPOSE? ☐ USED AS HUNTING DOG ☐ ASSISTANCE DOG
☐ MOUSER ☐ OTHER: ____

11. HAS THE ANIMAL BEEN AWAY FROM ITS PLACE OF RESIDENCE DURING THE LAST TWO WEEKS? ☐ YES COUNTRY POSTAL CODE
☐ NO

# THANK YOU FOR PARTICIPATION!

For questions about the dispatch of the tick kit
please send an e-mail to
info@zeckenkit.de

MSD Tiergesundheit

STIFTUNG TIERÄRZTLICHE HOCHSCHULE HANNOVER 1778
