# Supplementary material for: Attachment sites of Ixodes ricinus, Ixodes hexagonus/Ixodes canisuga and Dermacentor reticulatus ticks and risk factors of infestation intensity and engorgement duration in dogs and cats
Source: BMC Vet Res. 2025 Feb 22;21:83. doi: 10.1186/s12917-025-04535-z (PMC11846248; doi:10.1186/s12917-025-04535-z)
Supplement: Supplementary file 2 — Supplementary Material 2 [file 12917_2025_4535_MOESM2_ESM.docx]

**Additional Table 1:** Number of *I. ricinus*, *D. reticulatus* and *I. hexagonus* / *I. canisuga* ticks collected from dogs and cats in the tick collection study according to their detailed site of attachment. ”All species” refers to the sum of all collected tick species, including other than the three most frequent species.

|  | **Attachment sites on dogs** | | | | **Attachment sites on cats** | | | |
| --- | --- | --- | --- | --- | --- | --- | --- | --- |
|  | **All species** | ***I. ricinus*** | ***D. reticulatus*** | ***I. hexagonus* / *I. canisuga*** | **All species** | ***I. ricinus*** | ***D. reticulatus*** | ***I. hexagonus* / *I. canisuga*** |
| **Head (without ears)** | 2,051/8,178 (25.1%) | 1,936/6,634 (29.2%) | 78/1,267 (6.2%) | 51/187 (27.3%) | 1,349/6,807 (19.8%) | 1,295/6,282 (20.6%) | 8/48 (16.7%) | 78/388 (20.1%) |
| Head (not specified) | 40 | 32 | 1 | 0 | 3 | 2 | 0 | 1 |
| Head dorsal | 880 | 822 | 37 | 11 | 606 | 540 | 2 | 46 |
| Head ventral | 152 | 133 | 14 | 4 | 262 | 240 | 6 | 13 |
| Forehead | 29 | 29 | 0 | 0 | 8 | 8 | 0 | 0 |
| Muzzle | 462 | 430 | 7 | 21 | 159 | 146 | 0 | 6 |
| Lips | 0 | 3 | 0 | 0 | 0 | 4 | 0 | 0 |
| Chin | 143 | 132 | 6 | 1 | 140 | 133 | 0 | 4 |
| Nose | 11 | 11 | 0 | 0 | 1 | 1 | 0 | 0 |
| Cheek | 225 | 207 | 8 | 8 | 134 | 124 | 0 | 6 |
| Tongue | 1 | 1 | 0 | 0 | 0 | 0 | 0 | 0 |
| Eyes | 108 | 136 | 5 | 6 | 36 | 97 | 0 | 2 |
| **Ears** | 832/8,178 (10.2%) | 690/6,634 (10.4%) | 53/1,267 (4.2%) | 37/187 (19.8%) | 889/6,807 (13.1%) | 734/6,282 (11.7%) | 3/48 (6.3%) | 78/388 (20.1%) |
| Ear (not specified) | 6 | 6 | 0 | 1 | 1 | 1 | 0 | 0 |
| Ear cranial | 319 | 273 | 18 | 23 | 205 | 161 | 0 | 38 |
| Ear caudal | 97 | 80 | 8 | 6 | 51 | 29 | 0 | 21 |
| In front of the ear | 58 | 54 | 0 | 3 | 35 | 35 | 0 | 0 |
| Behind the ear | 283 | 249 | 27 | 3 | 485 | 460 | 3 | 18 |
| Under the ear | 62 | 22 | 0 | 1 | 110 | 46 | 0 | 1 |
| In the ear | 5 | 4 | 0 | 0 | 1 | 1 | 0 | 0 |
| Ear base | 2 | 2 | 0 | 0 | 1 | 1 | 0 | 0 |
| **Neck** | 1,694/8,178 (20.7%) | 1,293/6,634 (19.5%) | 355/1,267 (28.0%) | 24/187 (12.8%) | 3,049/6,807 (44.8%) | 2,869/6,282 (45.7%) | 18/48 (37.5%) | 71/388 (18.3%) |
| Neck (not specified) | 8 | 0 | 0 | 0 | 63 | 0 | 0 | 0 |
| Neck dorsal | 472 | 354 | 105 | 5 | 911 | 875 | 6 | 22 |
| Neck ventral | 466 | 321 | 131 | 11 | 652 | 627 | 6 | 12 |
| Neck lateral | 574 | 467 | 99 | 5 | 1167 | 1128 | 6 | 24 |
| Cervical neck | 174 | 151 | 20 | 3 | 256 | 239 | 0 | 13 |
| **Rump** | 1,984/8,178 (24.3%) | 1,526/6,634 (23.0%) | 422/1,267 (33.3%) | 21/187 (11.2%) | 725/6,807 (10.7%) | 709/6,282 (11.3%) | 7/48 (14.6%) | 60/388 (15.5%) |
| Chest | 277 | 203 | 69 | 3 | 139 | 131 | 0 | 7 |
| Abdomen | 182 | 139 | 37 | 3 | 37 | 28 | 0 | 9 |
| Abdominal wall | 413 | 284 | 118 | 4 | 130 | 115 | 1 | 10 |
| Back | 429 | 302 | 119 | 5 | 202 | 178 | 2 | 18 |
| Axilla | 366 | 311 | 56 | 3 | 127 | 176 | 3 | 7 |
| Inguinal area | 181 | 163 | 12 | 3 | 23 | 21 | 1 | 1 |
| Inter-thigh area | 45 | 42 | 2 | 0 | 4 | 3 | 0 | 1 |
| Anogenital area | 91 | 82 | 9 | 0 | 63 | 56 | 0 | 7 |
| **Frontlegs** | 866/8,178 (10.6%) | 624/6,634 (9.4%) | 204/1,267 (16.1%) | 30/187 (16.0%) | 552/6,807 (8.1%) | 505/6,282 (8.0%) | 6/48 (12.5%) | 37/388 (9.5%) |
| Shoulder | 415 | 290 | 114 | 6 | 317 | 302 | 5 | 7 |
| Between the shoulders | 133 | 94 | 36 | 2 | 165 | 156 | 0 | 9 |
| Upper arm | 156 | 121 | 35 | 0 | 30 | 22 | 1 | 7 |
| Elbow | 12 | 12 | 0 | 0 | 0 | 0 | 0 | 0 |
| Elbow flexion | 19 | 19 | 0 | 0 | 2 | 1 | 0 | 1 |
| Front paw | 131 | 88 | 19 | 22 | 38 | 24 | 0 | 13 |
| **Hindlegs** | 652/8,178 (8.0%) | 505/6,634 (7.6%) | 117/1,267 (9.2%) | 24/187 (12.8%) | 149/6,807 (2.2%) | 106/6,282 (1.7%) | 4/48 (8.3%) | 38/388 (9.8%) |
| Hip | 120 | 82 | 35 | 1 | 33 | 22 | 1 | 9 |
| Upper thigh | 317 | 266 | 43 | 7 | 70 | 55 | 3 | 12 |
| Knee | 9 | 9 | 0 | 0 | 1 | 1 | 0 | 0 |
| Knee fold | 128 | 95 | 29 | 3 | 27 | 22 | 0 | 5 |
| Ankle joint | 18 | 14 | 3 | 0 | 3 | 2 | 0 | 1 |
| Back paw | 60 | 39 | 7 | 13 | 15 | 4 | 0 | 11 |
| **Other** | 99/8,178 (1.2%) | 60/6,634 (0.9%) | 38/1,267 (3.0%) | 0/187  (0.0%) | 94/6,807 (1.4%) | 64/6,282 (1.0%) | 2/48 (4.2%) | 26/388 (6.7%) |
| Tail | 39 | 22 | 17 | 0 | 25 | 15 | 0 | 10 |
| Tail base | 39 | 29 | 9 | 0 | 42 | 24 | 1 | 16 |
| Dorsal (not specified) | 1 | 0 | 1 | 0 | 27 | 25 | 1 | 0 |
| Ventral (not specified) | 20 | 9 | 11 | 0 | 0 | 0 | 0 | 0 |
| **Total** | **8,178** | **6,634** | **1,267** | **187** | **6,807** | **6,282** | **48** | **388** |
