# Supplementary material for: Attachment sites of Ixodes ricinus, Ixodes hexagonus/Ixodes canisuga and Dermacentor reticulatus ticks and risk factors of infestation intensity and engorgement duration in dogs and cats
Source: BMC Vet Res. 2025 Feb 22;21:83. doi: 10.1186/s12917-025-04535-z (PMC11846248; doi:10.1186/s12917-025-04535-z)
Supplement: Supplementary file 3 — Supplementary Material 3 [file 12917_2025_4535_MOESM3_ESM.docx]

**Additional Table 2:** Number of adult *Ixodes ricinus* ticks collected from cats during a clinical laboratory study according to their detailed site of attachment. Note that the cats wore a collar to prevent tick removal.

|  | ***I. ricinus* collected from experimentally infested cats** |
| --- | --- |
| **Head (without ears)** | 209/848 (24.7%) |
| Head lateral | 1 |
| Head dorsal | 49 |
| Head ventral | 21 |
| Forehead | 18 |
| Muzzle | 33 |
| Chin | 62 |
| Cheek | 21 |
| Eyes | 4 |
| **Ears** | 316/848 (37.3%) |
| Ear cranial | 74 |
| Ear caudal | 98 |
| In front of the ear | 87 |
| Behind the ear | 25 |
| In the ear | 32 |
| **Neck** | 242/848 (28.5%) |
| Neck dorsal | 78 |
| Neck ventral | 46 |
| Neck lateral | 69 |
| Cervical neck | 49 |
| **Rump** | 26/848 (3.1%) |
| Chest | 12 |
| Abdominal wall | 1 |
| Back | 8 |
| Axilla | 3 |
| Inguinal area | 1 |
| Anogenital area | 1 |
| **Frontlegs** | 18/848 (2.1%) |
| Shoulder | 8 |
| Between the shoulders | 3 |
| Front paw | 7 |
| **Hindlegs** | 4/848 (0.5%) |
| Upper thigh | 2 |
| Knee fold | 2 |
| **Tail base** | 29/848 (3.4%) |
| **Total** | 848 |
