# Supplementary material for: Attachment sites of Ixodes ricinus, Ixodes hexagonus/Ixodes canisuga and Dermacentor reticulatus ticks and risk factors of infestation intensity and engorgement duration in dogs and cats
Source: BMC Vet Res. 2025 Feb 22;21:83. doi: 10.1186/s12917-025-04535-z (PMC11846248; doi:10.1186/s12917-025-04535-z)
Supplement: Supplementary file 4 — Supplementary Material 4 [file 12917_2025_4535_MOESM4_ESM.docx]

**Additional Table 3:** Results of binominal GLMMs testing the influence of several predictor variables on the tick infestation type (single vs. multiple ticks) in dogs and cats for which breed information was available. The models were significantly different from null models containing only the dog respectively cat breed as a random factor (A: Chi-square = 109.21, Df = 26, *P* < 0.001; B: Chi-square = 152.37, Df = 20, *P* < 0.001). Significant *P*-values are printed in bold.

|  | Model A: Dogs (N = 3,684) | | | | Model B: Cats (N = 3,173) | | | |
| --- | --- | --- | --- | --- | --- | --- | --- | --- |
|  | Estimate | SE | z | *P* | Estimate | SE | z | *P* |
| Intercept | -2.67 | 0.67 | -3.997 | **< 0.001** | -1.80 | 0.76 | -2.366 | **0.018** |
| Age | **-** | **-** | **-** | **-** | -0.02 | 0.01 | -2.581 | **0.010** |
| Gender (ref: male) |  |  |  |  |  |  |  |  |
| Female | **-** | **-** | **-** | **-** | -0.15 | 0.08 | -1.803 | 0.071 |
| Height (ref: small) |  |  |  |  |  |  |  |  |
| Medium | 0.30 | 0.20 | 1.507 | 0.132 | **-** | **-** | **-** | **-** |
| Tall | 0.62 | 0.19 | 3.322 | **0.001** | -0.52 | 0.26 | -1.971 | **0.049** |
| Very tall | 0.51 | 0.22 | 2.261 | **0.024** | **-** | **-** | **-** | **-** |
| Coat length (ref: short) |  |  |  |  |  |  |  |  |
| Average | 0.17 | 0.13 | 1.285 | 0.199 | 0.40 | 0.44 | 0.905 | 0.366 |
| Long | 0.26 | 0.15 | 1.689 | 0.091 | 0.75 | 0.22 | 3.427 | **0.001** |
| Partially coated | 1.32 | 0.92 | 1.431 | 0.152 | **-** | **-** | **-** | **-** |
| Density of the undercoat (ref: missing) |  |  |  |  |  |  |  |  |
| Moderate | -0.05 | 0.18 | -0.261 | 0.794 | **-** | **-** | **-** | **-** |
| Dense | 0.26 | 0.14 | 1.953 | 0.051 | **-** | **-** | **-** | **-** |
| Structure of the hair coat (ref: straight) |  |  |  |  |  |  |  |  |
| Double coated hair | -0.05 | 0.15 | -0.325 | 0.745 | **-** | **-** | **-** | **-** |
| Wavy/Shaggy hair | 0.12 | 0.17 | 0.683 | 0.494 | **-** | **-** | **-** | **-** |
| Shape of the ears (ref: erect) |  |  |  |  |  |  |  |  |
| Folded* | 0.26 | 0.13 | 1.957 | **0.050** | **-** | **-** | **-** | **-** |
| Dropping | 0.22 | 0.25 | 0.871 | 0.384 | **-** | **-** | **-** | **-** |
| Character of residence (ref: rural) |  |  |  |  |  |  |  |  |
| Urban | -0.24 | 0.11 | -2.253 | **0.024** | -0.22 | 0.11 | -1.992 | **0.046** |
| Rural and urban | -0.12 | 0.15 | -0.810 | 0.418 | 0.01 | 0.25 | 0.039 | 0.969 |
| Only on the own property | **-** | **-** | **-** | **-** | -0.65 | 0.31 | -2.113 | **0.035** |
| Month of collection (ref: January) |  |  |  |  |  |  |  |  |
| February | 1.06 | 0.70 | 1.512 | 0.131 | 1.03 | 0.81 | 1.274 | 0.203 |
| March | 0.46 | 0.64 | 0.726 | 0.468 | 1.26 | 0.76 | 1.661 | 0.097 |
| April | 0.84 | 0.63 | 1.345 | 0.179 | 1.63 | 0.75 | 2.159 | **0.031** |
| May | 0.89 | 0.63 | 1.430 | 0.153 | 1.53 | 0.75 | 2.029 | **0.042** |
| June | 1.09 | 0.62 | 1.740 | 0.082 | 1.55 | 0.76 | 2.056 | **0.040** |
| July | 0.37 | 0.63 | 0.585 | 0.559 | 1.08 | 0.76 | 1.419 | 0.156 |
| August | 0.02 | 0.66 | 0.028 | 0.978 | 0.33 | 0.80 | 0.409 | 0.683 |
| September | 0.05 | 0.65 | 0.071 | 0.943 | 0.26 | 0.79 | 0.326 | 0.745 |
| October | 0.38 | 0.66 | 0.582 | 0.560 | 0.11 | 0.79 | 0.133 | 0.895 |
| November | -0.08 | 0.69 | -0.119 | 0.905 | -0.33 | 0.82 | -0.401 | 0.688 |
| December | 0.08 | 0.78 | 0.104 | 0.917 | 0.94 | 0.87 | 1.088 | 0.277 |
| Year of collection (ref: 2020) |  |  |  |  |  |  |  |  |
| 2021 | -0.44 | 0.11 | -3.843 | **< 0.001** | -0.19 | 0.13 | -1.494 | 0.135 |

SE = Standard Error
