# Supplementary material for: Attachment sites of Ixodes ricinus, Ixodes hexagonus/Ixodes canisuga and Dermacentor reticulatus ticks and risk factors of infestation intensity and engorgement duration in dogs and cats
Source: BMC Vet Res. 2025 Feb 22;21:83. doi: 10.1186/s12917-025-04535-z (PMC11846248; doi:10.1186/s12917-025-04535-z)
Supplement: Supplementary file 5 — Supplementary Material 5 [file 12917_2025_4535_MOESM5_ESM.docx]

**Additional Table 4:** Results of binominal GLMMs testing the influence of several predictor variables on the duration of engorgement (≤ 48 hrs vs. > 48 hrs) of ticks collected from dogs and cats. The models were significantly different from null models containing the dog breed and animal ID as a random factor for ticks from dogs and only the ID for ticks from cats (A: Chi-square = 78.17, Df = 15, *P* < 0.001; B: Chi-square = 33.69, Df = 13, *P* = 0.001). Significant *P*-values are printed in bold.

|  | Model A: Ticks from dogs (N = 2,101) | | | | Model B: Ticks from cats (N = 2,583) | | | |
| --- | --- | --- | --- | --- | --- | --- | --- | --- |
|  | Estimate | SE | z | *P* | Estimate | SE | z | *P* |
| Intercept | 1.52 | 0.26 | 5.957 | **< 0.001** | 1.16 | 0.27 | 4.253 | **< 0.001** |
| Age group (ref: adult) |  |  |  |  |  |  |  |  |
| Juvenile | -0.11 | 0.16 | -0.694 | 0.487 | 0.12 | 0.15 | 0.751 | 0.453 |
| Young adult | -0.39 | 0.18 | -2.162 | **0.031** | 0.09 | 0.12 | 0.718 | 0.473 |
| Senior | 0.45 | 0.17 | 2.727 | **0.006** | 0.54 | 0.17 | 3.123 | **0.002** |
| Gender (ref: male) |  |  |  |  |  |  |  |  |
| Female | -0.08 | 0.12 | -0.672 | 0.502 | 0.30 | 0.10 | 2.937 | **0.003** |
| Body size (ref: medium) |  |  |  |  |  |  |  |  |
| Tall | **-** | **-** | **-** | **-** | -0.55 | 0.38 | -1.452 | 0.146 |
| Coat length (ref: short) |  |  |  |  |  |  |  |  |
| Average | 0.03 | 0.15 | 0.173 | 0.863 | -0.23 | 0.61 | -0.372 | 0.710 |
| Long | -0.01 | 0.18 | -0.040 | 0.968 | 0.23 | 0.36 | 0.648 | 0.517 |
| Partially coated | -1.43 | 1.15 | -1.238 | 0.216 | **-** | **-** | **-** | **-** |
| Density of the undercoat (ref: dense) |  |  |  |  |  |  |  |  |
| Missing | 0.19 | 0.17 | 1.134 | 0.257 | -0.10 | 0.52 | -0.187 | 0.852 |
| Moderate | -0.15 | 0.21 | -0.710 | 0.478 | 0.13 | 0.25 | 0.498 | 0.618 |
| Breed purpose (ref: companion breeds) |  |  |  |  |  |  |  |  |
| Toy breeds | 0.03 | 0.23 | 0.119 | 0.905 | **-** | **-** | **-** | **-** |
| Hunting breeds | -0.03 | 0.19 | -0.153 | 0.878 | **-** | **-** | **-** | **-** |
| Working and utility breeds | 0.51 | 0.24 | 2.120 | **0.034** | **-** | **-** | **-** | **-** |
| Herding breeds | 0.36 | 0.24 | 1.501 | 0.133 | **-** | **-** | **-** | **-** |
| Greyhounds | -0.65 | 0.51 | -1.282 | 0.200 | **-** | **-** | **-** | **-** |
| Animal infestation intensity | -0.14 | 0.03 | -5.582 | **< 0.001** | 0.01 | 0.01 | 0.619 | 0.536 |
| Character of residence (ref: rural) |  |  |  |  |  |  |  |  |
| Urban | **-** | **-** | **-** | **-** | 0.00 | 0.13 | 0.015 | 0.988 |
| Rural and urban | **-** | **-** | **-** | **-** | -0.75 | 0.28 | -2.663 | **0.008** |
| Only on the own property | **-** | **-** | **-** | **-** | 0.01 | 0.34 | 0.016 | 0.987 |

SE = Standard Error
